# Improving Ablation Safety for Hepatocellular Carcinoma Proximal to the Hilar Bile Ducts by Ultrasound-MR Fusion Imaging: A Preliminary Comparative Study

**DOI:** 10.3389/fonc.2021.570312

**Published:** 2021-03-01

**Authors:** Yujia You, Yinglin Long, Ronghua Yan, Liping Luo, Man Zhang, Lu Li, Qingjing Zeng, Kai Li, Rongqin Zheng, Erjiao Xu

**Affiliations:** ^1^Department of Ultrasound, The Third Affiliated Hospital of Sun Yat-Sen University, Guangzhou, China; ^2^Department of Radiology, The Eighth Affiliated Hospital of Sun Yat-Sen University, Shenzhen, China; ^3^Department of Medical Ultrasonics, The Eighth Affiliated Hospital of Sun Yat-Sen University, Shenzhen, China

**Keywords:** hepatocellular carcinoma (HCC), bile duct, radiofrequency ablation, safety, ultrasonography

## Abstract

**Aim:**

To explore whether ablation safety could be improved by ultrasound (US)-magnetic resonance (MR) fusion imaging for hepatocellular carcinoma (HCC) proximal to the hilar bile ducts (HBDs) through a preliminary comparative study.

**Methods:**

Between January 2014 and June 2019, 18 HCC nodules proximal to the HBDs were included in a US-MR fusion imaging-assisted radiofrequency ablation (RFA) group (study group), while 13 HCC nodules in a similar location were included as a control group. For the study group, the tumor and adjacent bile ducts were outlined on preprocedural MR images. Procedural ablation planning was conducted to assess the feasibility of ablating the tumors while avoiding biliary injury. Such tumors were then ablated under US-MR fusion imaging guidance. The control group nodules were ablated under conventional ultrasound guidance. Baseline characteristics and outcomes were compared between the groups.

**Results:**

After preprocedural assessment, 14 of 18 patients with tumors that were feasible to ablate underwent US-MR fusion imaging-assisted RFA. No biliary complications were observed in these 14 patients; the complication rate was significantly lower in the study group than in the control group (30.8%, 4/13) (*P* = 0.041). There was no significant difference in the technique efficacy rates [92.9% (13/14) *versus* 100% (13/13), *P* = 1] or local progression rates [7.1% (1/14) *versus* 7.7% (1/13), *P* = 1] between the study and control groups.

**Conclusions:**

US-MR fusion imaging may be a non-invasive means for assisting RFA of HCC nodules proximal to the HBDs and ensuring ablation safety.

## Introduction

Radiofrequency ablation (RFA) has been recommended as one of the first-line treatment options for early- or very-early-stage hepatocellular carcinoma (HCC) because this procedure is effective, minimally invasive, and safe (1, 2). However, an HCC nodule location proximal to the hilar bile ducts (HBDs) is considered to be a relative contraindication for RFA since the hilar bile ducts are vulnerable to thermal damage (3, 4). Ablation-related biliary complications (such as bile leakage, biliary stricture and obstruction) occurred in up to 31–46% of patients in previous reports (5, 6); such complications may prolong hospitalization, increase the cost of treatment, and even lead to mortality (7). Since the bile ducts are usually difficult to discern on ultrasound (US) images (the most common guidance tool used for RFA of HCC), this may increase the risk of thermal damage to the bile ducts during US-guided ablation. On the other hand, when operators tend to restrict the ablation area to reduce thermal damage to the HBDs, the reduced complications may be accompanied by higher incidences of residual tumor or local tumor progression (LTP) (8).

To ablate nodules proximal to the HBDs as completely as possible and reduce the risk of thermal biliary damage, several strategies have been employed. Percutaneous ethanol injection (PEI) or transarterial chemoembolization (TACE) with or without RFA is generally used to treat HCC nodules proximal to the hilar bile ducts (9, 10). However, the therapeutic effect of PEI or TACE is limited compared to that of RFA. Another strategy is intraductal chilled saline perfusion through an endoscopic nasobiliary drainage (ENBD) tube or a percutaneous transhepatic cholangial drainage (PTCD) tube to cool the large bile duct during the RFA procedure (11–15). However, both catheterization procedures are invasive and may lead to unexpected pancreatitis. Moreover, percutaneous transhepatic cholangial drainage is usually technically difficult in patients with nondilated intrahepatic bile ducts.

Ultrasound (US)-computed tomography (CT)/magnetic resonance (MR) fusion imaging is a navigation technique that is increasing in popularity that simultaneously combines the real-time capacity of US imaging and the high spatial resolution of CT/MR imaging (16). US-CT/MR fusion imaging has been widely used during US-guided RFA procedures for HCC detection, guidance, and evaluation (17–24). A few studies have integrated the use of a three-dimensional ablation planning system for the ablation procedure for liver tumors to enhance the complete ablation rate and reduce operators’ experience dependence (25–27). However, there are no studies evaluating the advantages of ablation planning and fusion imaging for enhancing ablation safety for tumors in high-risk locations.

Since the course of the HBDs is usually more clearly outlined by MR-specific sequences (e.g., T2 or hepatobiliary-phase sequences) than by US or CT images (28), we hypothesize that US-MR fusion imaging (in which a planning module has been integrated) might be helpful during the RFA procedure for HCC nodules proximal to the HBDs. In the planning module, both the tumor and its adjacent HBDs can be outlined easily on MR images and then displayed three-dimensionally; meanwhile, their spatial relationship can be displayed automatically on real-time US images through US-MR fusion imaging. Then, the operator can make an ablation plan (including choosing a puncture path and presetting the electrode placements) and preview the simulated thermal fields to assess the risk of bile duct injury. In addition, the RFA procedure can be performed precisely under the guidance of US-MR fusion imaging according to the preprocedural plan.

Here, we aimed to explore whether ablation safety could be improved by US-MR fusion imaging for HCC nodules adjacent to the HBDs through a preliminary comparative study.

## Methods

### Study Design

From January 2014 to June 2019, patients with HCC nodules adjacent to the HBDs were prospectively enrolled into a US-MR fusion imaging-assisted group through a non-randomized study design. This study was approved by the institutional review board of our hospital. Written informed consent was obtained from each patient. To compare the safety of US-MR fusion imaging-assisted RFA for HCC with that of conventional US-guided RFA, patients with HCC nodules adjacent to the HBDs who previously underwent conventional US-guided RFA were retrospectively included as a control group.

The inclusion criteria were as follows: (1) pathological or clinical diagnosis of HCC (29); (2) tumor located within 10 mm proximal to the HBDs (referring to the left hepatic duct, right hepatic duct, and their conjunction); and (3) indications for RFA (29). The exclusion criteria were as follows: (1) patients who were unable or unwilling to participate in follow-up, and (2) patients with pacemakers.

### Equipment and Agents

#### US Machine

A MyLab 90 or MyLab Twice US machine (Esoate, Genoa, Italy) with a convex array probe (CA541, frequency range from 1 to 8 MHz) was used for imaging guidance. The Virtual Navigator (Esoate, Genoa, Italy) electromagnetic positioning system was the main unit of the US machine. A planning module was installed as a system component of the Virtual Navigator.

#### RFA System

A cool-tip radiofrequency (RF) generator (Covidien, Mansfield, USA) and an internally cooled electrode with a 30-mm tip were used in this study. The RF generator was set in impedance mode with a maximum output. The ablative time for each RF electrode insertion was approximately 4–12 min.

#### US Contrast Agents (UCAs)

SonoVue (Bracco, Milan, Italy) was used for contrast-enhanced US (CEUS). UCAs were injected as a rapid bolus of 1.0 to 2 ml *via* an antecubital vein, followed by 5 ml of saline solution.

### US-MR Fusion Imaging-Assisted RFA

US-MR fusion imaging-assisted RFA was performed by a senior interventional doctor (X.E.J.) with 10 years of experience with RFA and fusion imaging.

#### Preprocedural Evaluation of Ablation Feasibility Using US-MR Fusion Imaging-Based Planning

##### MR Image Preparation

**Figure 1:**
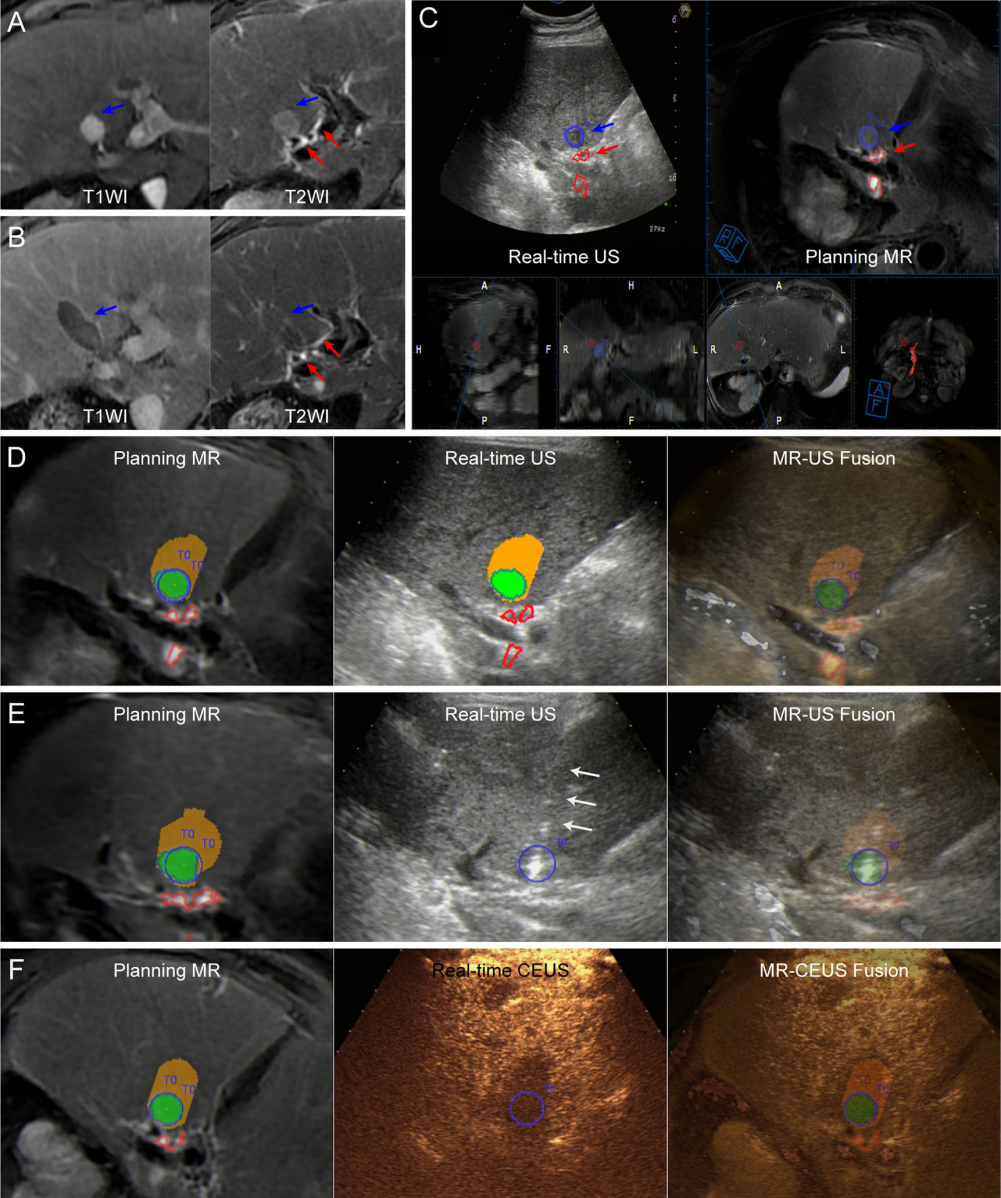
Images from a 72-year-old man diagnosed with HCC adjacent to the right bile duct. **(A)** Preoperative MR images showing the target tumor with a maximum diameter of 16 mm in segment 4 of the liver (blue arrow). Both the target tumor and adjacent bile ducts (red arrows) are displayed clearly on T2WI image (right). **(B)** At the 1-month follow-up MR, the index tumor (blue arrows) was completely ablated without injury to the adjacent bile ducts (red arrows). **(C)** Image depicting the target tumor and adjacent bile ducts. The tumor margins are outlined with red lines, while the ablative margins are outlined with yellow lines. **(D)** Assessment of ablation feasibility based on ablation planning. Simulated thermal fields (STP) are shown in yellow, and when the STP covered the tumor, the overlapping fields are shown in green. One simulated ablation was planned to cover 100% of the tumor volume without overlapping of adjacent bile ducts, and then the tumor was determined to have ablation feasibility. **(E)** Insertion of the electrode (white arrows) under the guidance of US-MR fusion imaging according to the ablation plan. **Supplementary Video 1** completely and dynamically shows the US-MR fusion imaging-guided insertion process. **(F)** Immediate evaluation with MR-CEUS fusion imaging after ablation. The tumor and ablative margins are overlapped by the non-perfusion area.

Digital image and communication on medicine (DICOM) data from T2-weighted or hepatobiliary phase MR sequences (**Figure 1A**) were selected routinely for US-MR fusion imaging due to clear visualization of the biliary tract and target tumor. DICOM data from MR images were imported into the Virtual Navigator.

##### Depiction of the Target Tumor and the HBDs

The target tumor was manually outlined in blue, and the course of the adjacent HBDs was depicted automatically or manually in red on the MR images (**Figure 1C**). The relationship between the marked tumor and the HBDs could be displayed three-dimensionally.

##### Registration of MR Images and Real-Time US Imaging

Registration was performed by preliminary registration and fine tuning for precise alignment between the MR images and real-time US images. The details of the registration process have been described in previous studies [10]. After successful registration, the MR images could be synchronized with the real-time US images (**Figure 1C**).

##### Preprocedural Evaluation of Ablation Feasibility

After the registration process, the three-dimensional spatial relationship between the target tumor and the course of the adjacent HBDs could be observed in different planes by moving the probe. The virtual thermal field of the RF electrode with the 30-mm tip was set as an ellipsoid 30 × 20 mm in size in the planning module. Preprocedural planning was carried out by the senior interventional US doctor (X.E.J.). After determination of the entry point on US-MR fusion imaging, one or multiple ellipsoids corresponding to the virtual thermal field were utilized to draft the electrode placement strategy (**Figure 1D**).

The aim of ablation planning was to achieve as much coverage of the target tumor as possible by the simulated thermal field (at least 90% volume) while avoiding coverage of the adjacent HBDs. In addition, in principle, ablations should be planned as few times as possible. Since the spatial relationship among the simulated thermal field, the target tumor and the adjacent bile duct could be observed by three-dimensional visualization, the operator could adjust the electrode placements conveniently to achieve the aim of planning. If the aim of planning could be obtained successfully within five adjustments, RFA was considered feasible. Otherwise, RFA alone was considered infeasible, and other treatment options were considered instead.

#### Implementation of RFA According to the Preprocedural Plan

All patients underwent RFA under endotracheal general anesthesia. Before implementation of RFA, the registration procedure with US-MR fusion imaging was performed again, and the preprocedural plan was reconfirmed in the operating theater. Subsequently, electrode insertion was performed following the preprocedural plan precisely according to the electrode placement strategy under the guidance of US-MR fusion imaging (**Figure 1E** and **Supplementary Video 1**).

During the RFA procedure, US-MR fusion imaging was used to monitor the hyperechoic area produced by RFA and to assess whether the adjacent HBDs depicted on MR images were covered by the hyperechoic area, aiming to reduce the risk of thermal damage to the bile ducts as much as possible.

#### Postprocedural Immediate Assessment of the Ablative Effect

Approximately 5–10 min after the procedure, UCAs were administered intravenously to perform CEUS, and CEUS-MR fusion imaging was used to evaluate whether the non-perfusion zone covered the whole target tumor (**Figure 1F**) and whether perfusion of the UCAs in the adjacent bile duct wall was normal. If incomplete ablation was demonstrated by CEUS-MR fusion imaging, supplementary ablation was instantly carried out to achieve complete ablation.

### Conventional US-Guided RFA

From January 2014 to June 2019, patients with tumors proximal to the HBDs were treated with the conventional RFA. The ablation procedures were performed by one of three doctors (Z.R.Q, X.E.J., L.K.) with 5 to 10 years of experience with RFA.

Conventional RFA was performed under US guidance, and multiple overlapping ablations were performed to achieve complete ablation. At the end of the ablation procedure, CEUS was used for immediate assessment of the ablative effect.

### Postprocedural Surveillance and Follow-Up

After the procedure, US and laboratory examinations were performed regularly to exclude early complications. Biliary tract-related symptoms and signs were also closely surveilled.

One month later, contrast-enhanced CT (CECT) or contrast-enhanced MR (CEMR) was performed to evaluate the technical efficacy and complications (**Figure 1B**). Then, follow-up imaging and laboratory examinations were repeated every 3 months. LTP and ablation-related biliary complications were recorded until December 31, 2019.

### Statistical Analysis

SPSS 22.0 (SPSS, Chicago, IL, USA) was used for statistical analysis. Continuous measurement data are presented as the mean ± standard deviation if the data were normally distributed or as the median (range) if the data were not normally distributed. Enumeration data are presented as percentages. Variables in the two independent groups were compared using the two-sample t-test or Mann-Whitney test for continuous variables and Pearson’s χ2 test or Fisher’s exact test for categorical variables, while matched data in the groups were compared using the paired t-test or Wilcoxon signed-rank test for continuous variables and the McNemar test for categorical variables. A *P* value less than 0.05 was considered statistically significant.

## Results

### Patients and Tumors

**Figure 2:**
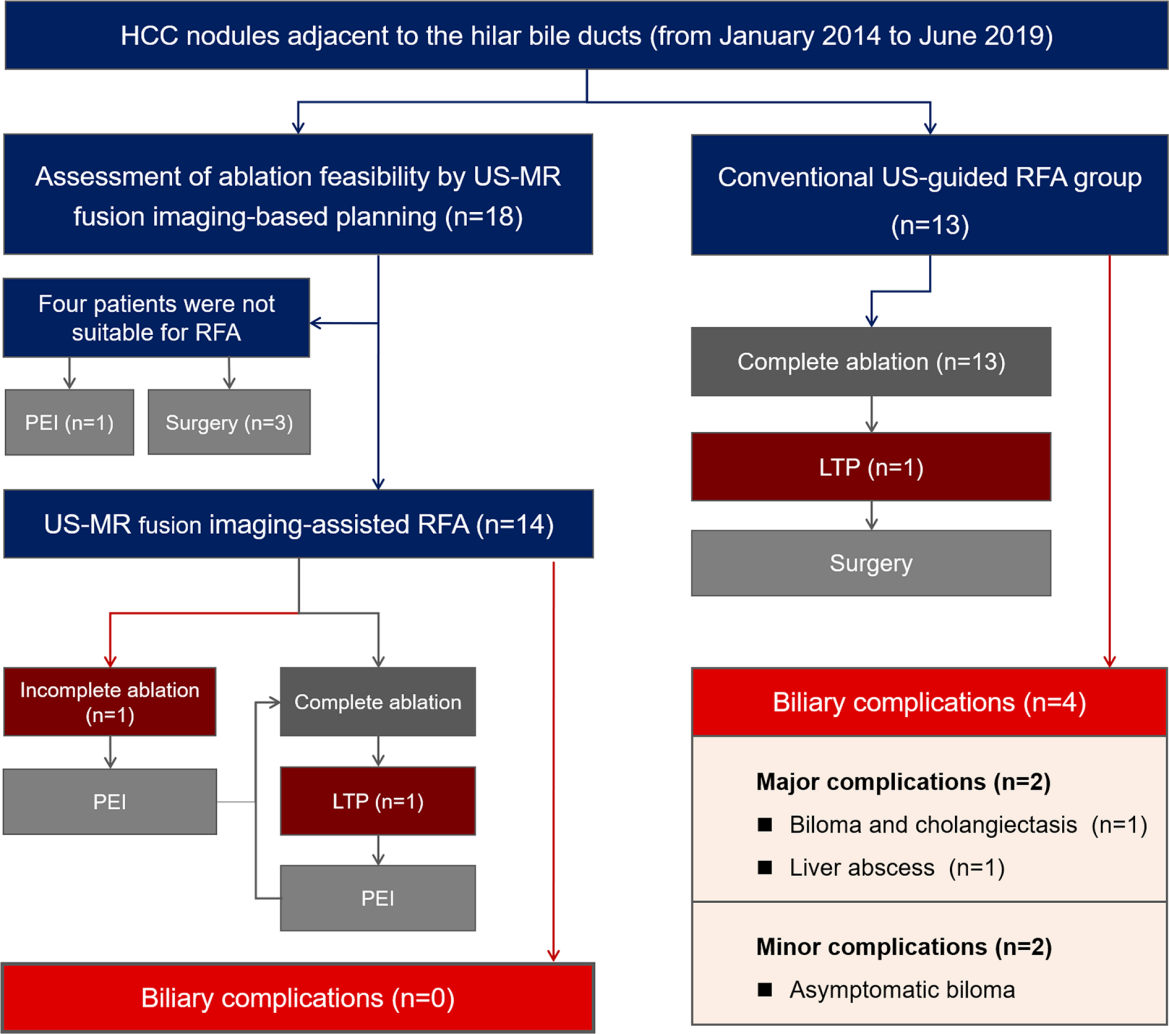
Overview of management and outcome of enrolled patients.

A total of 18 patients with 18 tumors proximal to the hilar bile ducts were enrolled in the US-MR fusion imaging-assisted RFA group. A total of 13 patients with 13 tumors were included in the control group. **Figure 2** provides a brief overview of these two groups in a flow chart. The baseline characteristics of the two groups are listed in **Table 1** and their detailed information are listed in **Tables S1** and **S2**.

**Table 1:** Baseline characteristics of enrolled patients and nodules in the US-MR fusion imaging-assisted RFA group and the conventional US-guided RFA group (control group).

Characteristics	US-MR fusion imaging-assisted RFA	Conventional US-guided RFA	*P* value
**Patient**	N = 14	N = 13	
Age	55 ± 9 (44–72)	53 ± 11 (35–69)	0.633
Gender (male/female)	14/0	11/2	0.222
Primary/recurrent	9/5	3/10	0.054
Diagnosed on pathological/clinical	11/3	5/8	0.054
Cirrhosis (yes/no)	12/2	7/6	0.103
Hepatitis virus infection (HBV/HCV/no)	13/1/0	12/0/1	0.367
AFP (≤200/>200)	13/1	9/4	0.165
PLT (/L)	163 ± 67 (82–279)	130 ± 39 (76–216)	0.139
PT (s)	14.1 ± 1.3 (12.3–17.8)	14.1 ± 1.2 (11.6–16.0)	0.991
ALB (g/L)	39.6 ± 5.8 (31.2–51.5)	40.8 ± 4.3 (35.5–50.2)	0.536
TBil (μmol/L)	14.7 ± 6.2 (7.7–28.5)	13.0 ± 5.6 (5.8–28.7)	0.470
Child Pugh Class (A/B)	13/1	13/0	1
**Nodules**	N = 14	N = 13	
Single/multiple	10/4	5/8	0.128
Location (left hemiliver/right hemiliver)	8/6	9/4	0.695
Maximum diameter (mm)	21.5 ± 11.2 (9–49)	18.7 ± 9.1 (11–43)	0.482
Adjacent bile duct (biliary confluence/left hepatic duct/right hepatic duct)	5/6/3	3/5/5	0.590
Distance between nodule and bile duct (mm)	**2.5 ± 1.9 (0–6)**	**5.9 ± 1.7 (4–9)**	**<0.001**
Distance <5 mm/5–10 mm	**12/2**	**4/9**	**0.006**

The values was shown in bold since their differences are statistically significant.

### US-MR Fusion Imaging-Assisted RFA

#### Preprocedural Evaluation of Ablation Feasibility With US-MR Fusion Imaging

US-MR fusion imaging registration was successful in all 18 patients. According to the preprocedural plan, four patients failed to achieve the aim of the electrode placement strategy, as coverage of the adjacent HBDs could not be avoided. The ablation feasibility assessment process in one of these four patients is given in **Figure S1** and **Supplementary Video 2**. Surgical resection was recommended for two patients, and the other two patients underwent PEI instead.

**Figure 3:**
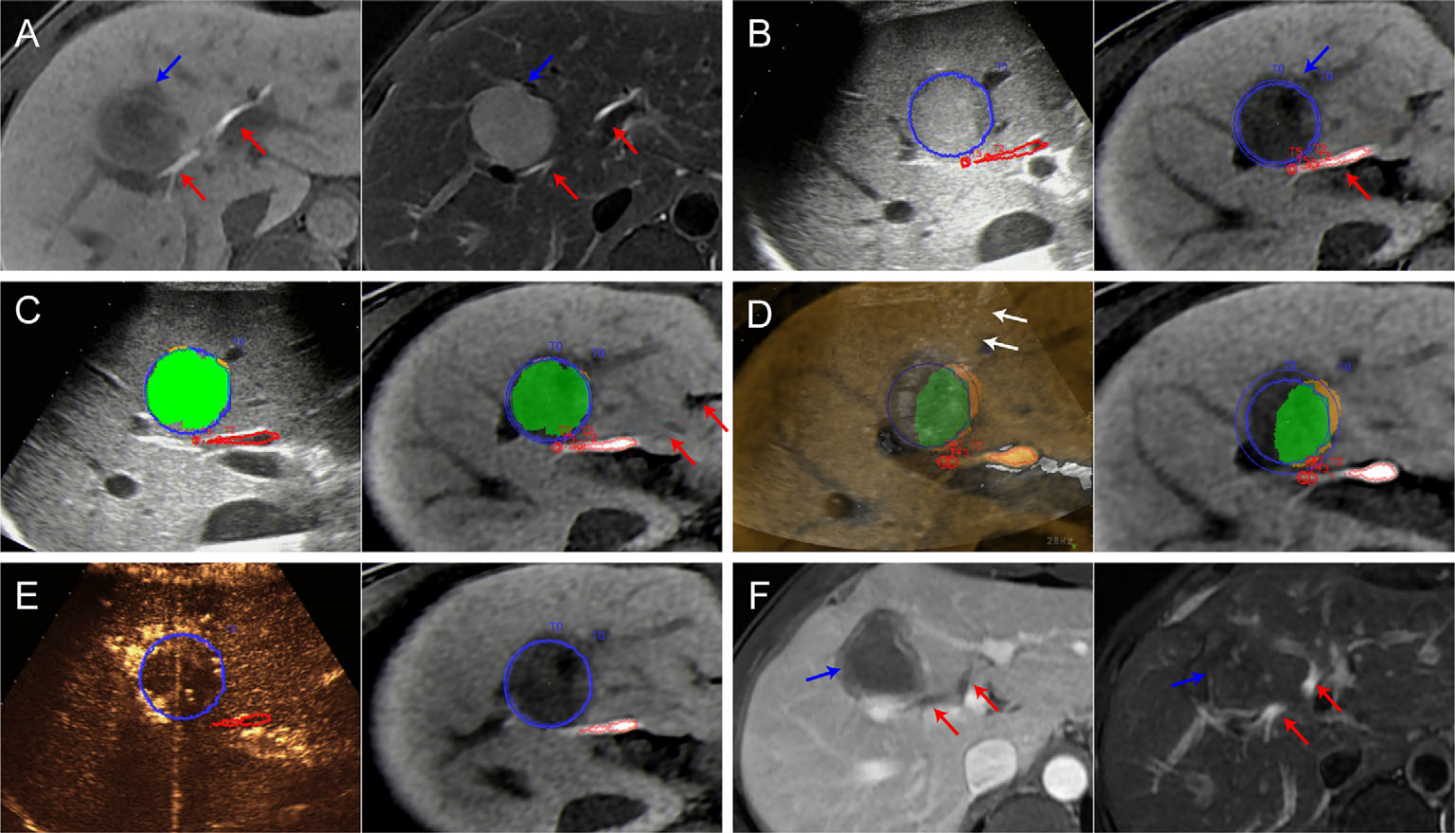
Images from a 44-year-old man diagnosed with HCC adjacent to the right bile duct. **(A)** Preoperative MR images showing the target tumor with a maximum diameter of 36 mm in segment 8 of the liver (blue arrow). Both the hepatobiliary phase (left) and T2WI images (right) clearly display the target tumor and adjacent bile ducts (red arrows). **(B)** Outline of the tumor and adjacent bile ducts. The tumor margins are outlined with red lines, and the ablative margins are outlined with yellow lines. **(C)** Ablation planning. Six simulated ablations were planned to cover 91% of the tumor volume. When the STP covered the tumor, the overlapping fields are presented in green. **(D)** Insertion of the radiofrequency electrode (white arrows) under the guidance of US-MR fusion imaging to implement the plan. **(E)** Immediate evaluation with MR-CEUS fusion imaging after ablation. The tumor and ablative margins are overlapped by the non-perfusion area. **(F)** At the 1-month follow-up MR, the index tumor (blue arrows) was completely ablated without injury to the adjacent bile ducts (red arrows).

In the remaining fourteen patients, RFA was considered feasible, and they underwent the procedure with US-MR fusion imaging guidance. **Figure 3** shows representative images of a patient who underwent US-MR fusion imaging-assisted ablation. The clinical characteristics of these 14 patients and nodules are presented in **Table 1**.

According to the preprocedural plan, 1 to 14 electrode placements (median: two times) were required to cover 90–100% (median: 100%) of the entire target nodule volume. The duration of preprocedural planning was 2–10 min (median: 5 min).

#### Implementation of RFA According to the Preprocedural Plan and Immediate Evaluation of Technique Success

Each electrode insertion was performed under the guidance of US-MR fusion imaging according to the preprocedural plan. Electrode insertion was performed 1 to 14 times (median: two times) in accordance with the preprocedural plan. The entire ablation procedure required 60–212 min (median: 82 min).

During the RFA procedure, the hyperechoic area was monitored using US-MR fusion imaging. In four of these patients, the ablation process was stopped earlier than planned to avoid the risk of thermal damage, since the hyperechoic area began to cover the marked course of the adjacent HBDs.

After the RFA procedure, the immediate assessment performed *via* CEUS-MR fusion imaging indicated that the non-perfusion zones covered the target nodule in all 14 patients. No supplementary ablation was needed in these patients. The perfusion of the course of the adjacent HBDs was normal during CEUS evaluation.

### Comparison of Safety Between the US-MR Fusion Imaging-Assisted RFA Group and the Control Group

#### Baseline Characteristics of the Control Group

The baseline characteristics of the control group are shown in **Figure 1**. When comparing the baseline characteristics between the control group and the study group, apart from the distance between the tumor and HBDs, no statistically significant differences were observed. The US-MR fusion imaging-assisted RFA group showed a significantly shorter distance between the nodule and the HBDs (2.5 ± 1.9 mm *versus* 5.9 ± 1.7 mm, *P* < 0.001) than the control group.

#### Comparisons of Safety and Efficacy

Comparisons of treatment-related results are shown in **Table 2**. The median follow-up periods in the US-MR fusion imaging-assisted RFA group and control group were 13 (range: 7–30) and 32 (range: 3–50) months respectively. During the follow-up period, no biliary complications occurred in the study group. No intrahepatic bile ducts were evidently dilated on follow-up imaging examinations. In the control group, four patients were diagnosed with biliary complications (including two major biliary complications and two minor biliary complications). The biliary complication rate in the control group was significantly higher than that in the study group [30.77% (4/13) *vs* 0% (0/14), *P* = 0.041].

**Table 2:** Comparisons of treatment-related results between the US-MR fusion imaging-assisted RFA group and the conventional US-guided RFA group.

Characteristics	US-MR fusion imaging-assisted RFA	Conventional US-guided RFA	*P* value
Number of ablations	2 (1–14)	3 (1–10)	0.195
Duration of ablation procedure (min)	89 (60–212)	105 (50–315)	0.923
Biliary complications (yes/no)	**0/14**	**4/9**	**0.041**
Major biliary complications	0	2	
Minor biliary complications	0	2	
Complete ablation (yes/no)	13/1	13/0	1
Local tumor progression (yes/no)	1/13	1/12	1

The values was shown in bold since their differences are statistically significant.

The two patients with major biliary complications were as follows: one patient was diagnosed with a liver abscess and bile fistula several days after the procedure; the other patient was diagnosed with a large biloma (with a maximum diameter of 55 mm) and intrahepatic bile duct dilation 1 month after the procedure. Multiple treatments, such as antibiotic therapy, percutaneous catheter drainage, and surgery, were performed to manage these complications. Two patients with asymptomatic bilomas were diagnosed 1 and 4 months after the procedure. Both of them were not treated and then classified as minor biliary complications.

According to the 1-month follow-up CECT/CEMR, residual tumor was detected in one patient (7.1%) in the study group and in none of the patients in the control group. There was no significant difference between the two groups. In this patient, the tumor could not be eradicated despite several courses of TACE. During the follow-up period, the LTP rate was 7.1% (1/14) in the study group and 7.7% (1/13) in the control group. These two nodules showing LTP were treated with PEI.

## Discussion

HCC located in the hepatic hilum has always been a dilemma when considering thermal ablation (11). Even intraductal cooling has been used to assist in ablation of tumors adjacent to the HBDs and to reduce biliary complications; however, this is still an invasive procedure. Here, we proposed a novel non-invasive method (US-MR fusion imaging) to assist the ablation procedure that resulted in satisfactory therapeutic outcomes. The technical efficacy and local tumor recurrence rates of US-MR fusion imaging-assisted RFA are comparable to those in previous reports (5, 10, 11) (technical efficacy rate, 80.0–100.0%; local tumor recurrence rate, 11.8–21.0%) of thermal ablation performed with invasive ancillary procedures, such as intraductal chilled saline perfusion. More importantly, no biliary complications were observed during the follow-up period. Furthermore, patients who underwent traditional US-guided RFA for HCC nodules in the similar location were enrolled as controls for preliminary comparisons. According to the data analysis, the US-MR fusion imaging-assisted RFA group showed a significantly lower biliary complication rate (0 *versus* 30.77%, *P* = 0.041) than the control group.

It’s worth noting that the distance between the tumor and adjacent hilar bile ducts has highly important consequences on the therapeutic effect of RFA. Lin et al. reported that a distance from the bile duct within 10 mm was thought to be a high-risk factor for ablation-related biliary complications for HCC nodules (30). RFA is still not recommended for application in HCC nodules adjacent to the HBDs in some clinical treatment guidelines of HCC (29). Theoretically, the injury risk increases with a shortened distance from the hilar bile duct. However, in this study the US-MR fusion imaging-assisted RFA group showed a lower biliary complication rate with a significantly shorter distance between the nodule and the HBDs (2.5 ± 1.9 mm *versus* 5.9 ± 1.7 mm, *P* < 0.001) than the control group. These data showed an increasing number of patients with HCC nodules with a short distance (≤5 mm) from the HBDs underwent RFA, indicating the improved safety with the assistance of US-MR fusion imaging.

The following characteristics of US-MR fusion imaging-assisted RFA contribute to its high ablation safety. First, MR-specific sequences (T2 or hepatobiliary phase sequences) were chosen as reference images for fusion imaging since the target tumor and adjacent HBDs could be displayed clearly on these images (31). Compared with previous reports that used ablation planning based on CT or US images (25, 26), MR has a distinct advantage in displaying the bile ducts. Moreover, after the target tumor and adjacent HBDs were depicted, the outlined structures could be displayed through three-dimensional visualization, to help the operator better understand the spatial relationship between the target tumor and the adjacent HBDs. Second, when performing preprocedure ablation planning, the ablation feasibility of the target tumor could be determined by evaluating the spatial relationship among the simulated thermal field, the target tumor, and the adjacent HBDs. This planning step facilitated the establishment of reasonable and individualized treatment for patients. For tumors that could not be covered by the simulated thermal field without covering the HBDs, RFA alone was considered with a high injury risk. As a result, the treatment strategy was adjusted as soon as possible. Other means (such as surgery, RFA combined with PEI) may need to be performed to reduce potential biliary complications.

In addition, US-MR fusion imaging played a crucial role in the implementation of the ablation plan, including guiding electrode insertions, monitoring ablation process, and assessing treatment response and bile duct blood supply. With the guidance of US-MR fusion imaging, the electrode could be precisely inserted into the liver according to the planned angle and depth, which would not be affected by the hyperechoic region generated by RFA. With the real-time monitoring of US-MR fusion imaging, the ablation duration could be adjusted timely and flexibly to avoid the injury of the hilar bile ducts. With immediate treatment response assessment of US-MR fusion imaging, it can be timely determined whether supplementary ablation should be needed at the end of the procedure, helping reduce the possibility of residual tumors.

Overall, compared with traditional US-guided ablation, the use of US-MR fusion imaging in multiple links throughout the whole procedure brought changes in many aspects such as the identification of bile ducts, the evaluation method of ablation feasibility, electrode placement strategy, and duration of each ablation. All of these complex factors are conductive to the improved safety in US-MR fusion imaging-assisted ablation.

Apart from the use of fusion imaging, it must be acknowledged that experience may also be a factor that contributes to the favorable results of the study group. However, even the experienced doctors could not have much confidence when ablating the nodules adjacent to the hilar bile ducts which could result in serious complications. US-MR fusion imaging can help the operator assess the ablation feasibility with objective and graphic planning processes other than subjective judgment based on their own experiences, and meanwhile precisely guide the implementation of the ablation planning. Both experienced and inexperienced operators could enhance their confidence in performing ablation and benefit from this technique. Before US-MR fusion imaging was introduced, PEI or combination therapy rather than RFA alone was preferred to the treatment of HCC nodules with a distance ≤5 mm from the HBDs in our center. With the assistance of US-MR fusion imaging and increased operator confidence of performing RFA, an increasing number of HCC nodules proximal to the HBDs could be candidates for RFA. These changes in the treatment strategy brought about by the US-MR fusion imaging-assisted RFA hold the potential to help extend the ablation indications for HCC nodules adjacent to the HBDs in current clinical guidelines.

In this study, RFA was employed in all patients because the thermal field of RFA was well controlled and precise for ablation planning and implementation (32, 33). However, there was one case of residual tumor, possibly because of a registration error occurring when the respiratory phase for fusion during the procedure was inconsistent with that during the registration process (34, 35). To achieve more precise fusion imaging planning and guidance, respiratory phase control should be considered in detail.

The main limitation of our study is that it is a single-center study with a small sample size. After all, early-stage HCC nodules proximal to the hepatic hilum are relatively rare and not frequently referred for US-guided RFA. Another limitation is the short follow-up period of the study group. A larger sample of patients and long-term follow-up are necessary. In addition, US-CT/MR fusion imaging requires high proficiency to ensure the precision of registration, and the planning module is only available in some specific US machines, so popularization is still difficult. However, with the increased attention of fusion imaging, this strategy may be increasingly recognized.

In conclusion, US-MR fusion imaging could be a non-invasive means for assisting in RFA for HCC nodules proximal to the HBDs to ensure ablation safety.

## Data Availability Statement

The raw data supporting the conclusions of this article will be made available by the authors, without undue reservation.

## Ethics Statement

The studies involving human participants were reviewed and approved by the institutional review board of Third Affiliated Hospital of Sun Yat-sen University. The patients/participants provided their written informed consent to participate in this study.

## Author Contributions

RZ and EX contributed to the study design. RY, LPL, MZ, LL, QZ, and KL contributed to data collection. YY and YL performed the statistical analyses and wrote the manuscript. All authors contributed to the article and approved the submitted version.

## Funding

This work was supported by the National Key Research and Development Program of China under Grant No. 2017YFC0112000; the National Natural Science Foundation of China under Grant No. 81401434; the Science and Technology Planning Project of Guangdong Province, China under Grant Nos. 2017A020215082, 2017A020215137, and 2017B090901034; the Science and Technology Planning Project of Guangzhou, China under Grant No. 201704020164; the Fundamental Research Funds for the Central Universities, China under Grant Nos. 18ykpy05 and 20ykpy37; and Health public welfare scientific research project in Futian District Shenzhen under Grant No. FTWS2020022.

## Conflict of Interest

The authors declare that the research was conducted in the absence of any commercial or financial relationships that could be construed as a potential conflict of interest.
