# Supplementary material for: Improving Ablation Safety for Hepatocellular Carcinoma Proximal to the Hilar Bile Ducts by Ultrasound-MR Fusion Imaging: A Preliminary Comparative Study
Source: Front Oncol. 2021 Mar 1;11:570312. doi: 10.3389/fonc.2021.570312 (PMC7957055; doi:10.3389/fonc.2021.570312)
Supplement: Supplementary file 1 [file DataSheet_1.docx]

Supplementary Materials

# Supplementary Tables

**Table S1.** Basic characteristics and outcomes of patients in the US-MR fusion-assisted RFA group.

| Patient No. | Gender  (F=Female;  M=Male) | Age | Child-Pugh class | Maximum diameter (mm) | Distance between the tumor and adjacent bile duct (mm) | Complete ablation (yes/no) | Local tumor progression(yes/no) | Biliary complications  (yes/no) |
| --- | --- | --- | --- | --- | --- | --- | --- | --- |
| 1 | M | 55 | A | 14 | 5 | Y | Y | N |
| 2 | M | 48 | A | 12 | 2 | Y | N | N |
| 3 | M | 51 | A | 18 | 3 | Y | N | N |
| 4 | M | 52 | A | 23 | 3 | N | N | N |
| 5 | M | 70 | A | 49 | 6 | Y | N | N |
| 6 | M | 72 | A | 16 | 2 | Y | N | N |
| 7 | M | 60 | B | 19 | 3 | Y | N | N |
| 8 | M | 48 | A | 11 | 2 | Y | N | N |
| 9 | M | 44 | A | 36 | 0 | Y | N | N |
| 10 | M | 53 | A | 19 | 4 | Y | N | N |
| 11 | M | 48 | A | 9 | 0 | Y | N | N |
| 12 | M | 55 | A | 35 | 0 | Y | N | N |
| 13 | M | 63 | A | 19 | 1 | Y | N | N |
| 14 | M | 47 | A | 21 | 4 | Y | N | N |

**Table S2.** Basic characteristics and outcomes of patients in the US-guided RFA group.

| Patient No. | Gender  (F=Female;  M=Male) | Age | Child-Pugh class | Maximum diameter (mm) | Distance between the tumor and adjacent bile duct (mm) | Complete ablation (yes/no) | Local tumor progression(yes/no) | Biliary complications  (yes/no) |
| --- | --- | --- | --- | --- | --- | --- | --- | --- |
| 1 | M | 42 | A | 18 | 7 | Y | N | Y |
| 2 | M | 58 | A | 18 | 9 | Y | N | N |
| 3 | M | 49 | A | 12 | 4 | Y | N | N |
| 4 | M | 61 | A | 14 | 4 | Y | N | N |
| 5 | M | 66 | A | 17 | 4 | Y | N | N |
| 6 | M | 42 | A | 15 | 6 | Y | Y | Y |
| 7 | F | 47 | A | 27 | 5 | Y | N | N |
| 8 | M | 38 | A | 14 | 8 | Y | N | Y |
| 9 | M | 62 | A | 15 | 7 | Y | N | Y |
| 10 | F | 64 | A | 11 | 6 | Y | N | N |
| 11 | M | 54 | A | 28 | 4 | Y | N | N |
| 12 | M | 35 | A | 11 | 6 | Y | N | N |
| 13 | M | 69 | A | 43 | 7 | Y | N | N |

# Supplementary Figures

**
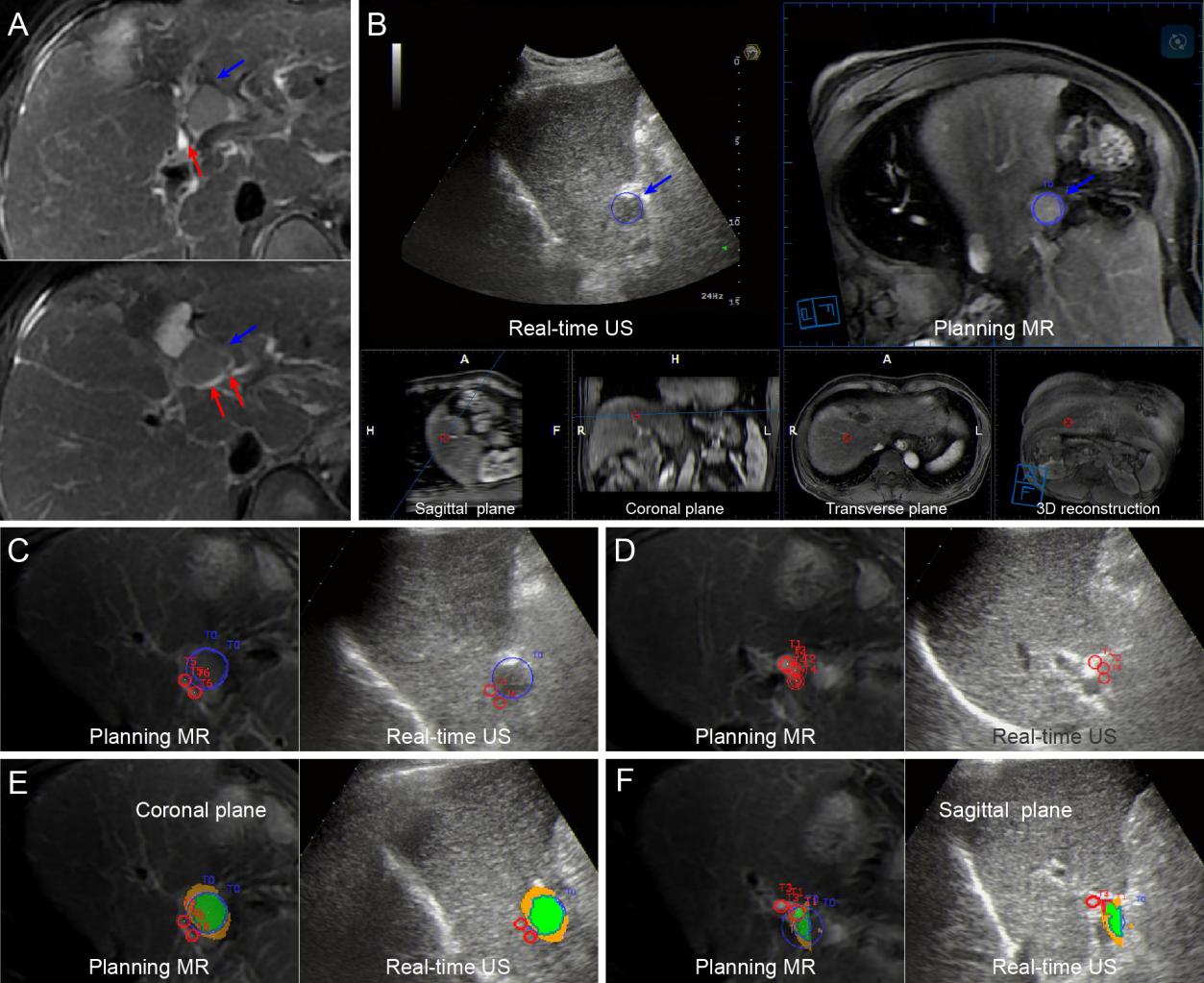
**

**Supplementary Figure 1.** Preprocedural assessment of ablation feasibility process in a 56-year-old man diagnosed with HCC adjacent to the left bile duct. **(A)** Preoperative MR images showing the target tumor with a maximum diameter of 20 mm in segment 4 of the liver (blue arrow). **(B)** Interface of ablation planning system (APS) based on US-MR fusion imaging. The tumor was outlined with red lines via the MR images, and the blue lines was synchronously displayed on the real-time US imaging. **(C)** and **(D)** shows the target tumor (blue circles) and adjacent bile ducts (marked with several red circles) in different planes. **(E)** and **(F)** assess the ablation feasibility via fusion imaging-based APS. Simulated thermal fields (STP) presented as yellow colour, when STP covered the tumor or bile ducts, the overlapping fields presented as green colour. In the plane shown in (F), STP adjustments were performed but failed to avoid overlapping of the adjacent bile ducts. Supplementary video 2 shows the complete spatial relationship between STP and marked bile ducts in multiple planes.

# Supplementary Videos

**Supplementary Video 1.** US-MR fusion imaging-guided electrode insertion process.

**Supplementary Video 2.** Preprocedural assessment of ablation feasibility via US-MR fusion imaging.
